# LatLRR-FCNs: Latent Low-Rank Representation With Fully Convolutional Networks for Medical Image Fusion

**DOI:** 10.3389/fnins.2020.615435

**Published:** 2021-01-13

**Authors:** Zhengyuan Xu, Wentao Xiang, Songsheng Zhu, Rui Zeng, Cesar Marquez-Chin, Zhen Chen, Xianqing Chen, Bin Liu, Jianqing Li

**Affiliations:** ^1^The Key Laboratory of Clinical and Medical Engineering, School of Biomedical Engineering and Informatics, Nanjing Medical University, Nanjing, China; ^2^The Department of Medical Engineering, Wannan Medical College, Wuhu, China; ^3^The Brain and Mind Centre, The University of Sydney, Sydney, NSW, Australia; ^4^The KITE Research Institute, Toronto Rehabilitation Institute-University Health Network, Toronto, ON, Canada; ^5^The Department of Electrical Engineering, College of Engineering, Zhejiang Normal University, Jinhua, China

**Keywords:** multi-modality medical image, latent low-rank representation, fully convolutional networks, medical image fusion, Laplacian pyramid

## Abstract

Medical image fusion, which aims to derive complementary information from multi-modality medical images, plays an important role in many clinical applications, such as medical diagnostics and treatment. We propose the LatLRR-FCNs, which is a hybrid medical image fusion framework consisting of the latent low-rank representation (LatLRR) and the fully convolutional networks (FCNs). Specifically, the LatLRR module is used to decompose the multi-modality medical images into low-rank and saliency components, which can provide fine-grained details and preserve energies, respectively. The FCN module aims to preserve both global and local information by generating the weighting maps for each modality image. The final weighting map is obtained using the weighted local energy and the weighted sum of the eight-neighborhood-based modified Laplacian method. The fused low-rank component is generated by combining the low-rank components of each modality image according to the guidance provided by the final weighting map within pyramid-based fusion. A simple sum strategy is used for the saliency components. The usefulness and efficiency of the proposed framework are thoroughly evaluated on four medical image fusion tasks, including computed tomography (CT) and magnetic resonance (MR), T1- and T2-weighted MR, positron emission tomography and MR, and single-photon emission CT and MR. The results demonstrate that by leveraging the LatLRR for image detail extraction and the FCNs for global and local information description, we can achieve performance superior to the state-of-the-art methods in terms of both objective assessment and visual quality in some cases. Furthermore, our method has a competitive performance in terms of computational costs compared to other baselines.

## 1. Introduction

Medical image fusion is a key technology that has been used extensively in clinical diagnosis and treatment planning (James and Dasarathy, 2014). Modern medical imaging techniques mainly include computed tomography (CT), magnetic resonance (MR), single-photon emission computed tomography (SPECT), and positron emission tomography (PET) (Walrand et al., 2017). CT has a high spatial and density resolution for dense structures (e.g., bones, implants), while MR has a high resolution for soft tissue (Wang et al., 2016) (e.g., muscle, tendon, and fascia). PET is an advanced nuclear medical examination technique that allows visualization of biomolecular metabolism, receptors, and neurotransmitter activity *in vivo*. SPECT is often applied to quantify images of the physiological and pathological changes of organs or tissues *in vitro*. Evaluating different perspectives of these imaging techniques reveals that they do, to an extent, complement each other (Walrand et al., 2017). Thus, medical image fusion can be utilized to combine different medical images and generate a new fusing image, providing the clinical information from each original image (Du et al., 2016; Huang et al., 2020).

To date, many medical image fusion studies have been reported (Toet, 1989; Li et al., 1995, 2013; Petrovic and Xydeas, 2004; Lewis et al., 2007; Zhang and Guo, 2009; Bhatnagar et al., 2015; Wang Q. et al., 2015; Geng et al., 2017; Zhao and Lu, 2017; Li H. et al., 2018; Manchanda and Sharma, 2018). Among them, multiscale transform (MST)-based methods are commonly used. The key point of MST-based fusion techniques is to decompose the original images into a multiscale transform domain (Li et al., 1995). Some fusion rule strategies can be utilized to merge the transformed coefficients, and the merged coefficients are employed to reconstruct the composite image. Note that the current literature indicates that the non-subsampled shearlet transform (NSST) and non-subsampled contourlet transform (NSCT) achieve the optimum performance in terms of image representation among MST-based methods (Anitha et al., 2015; Li Y. et al., 2018; Yin et al., 2018; Zhu et al., 2019). Zhu et al. used NSCT to decompose medical image pairs into low-pass and high-pass sub-bands, where a phase congruency rule was applied to fuse the high-pass sub-bands and a local Laplacian energy-based fusion rule was utilized for the low-pass sub-bands (Zhu et al., 2019). Later, Yin et al. introduced a novel framework in which the high-frequency coefficients were fused by a parameter-adaptive pulse coupled neural network (PA-PCNN), and the weighted local energy and the weighted sum of eight-neighborhood-based modified Laplacian were utilized to fuse low-frequency bands in the NSST domain (Yin et al., 2018). However, due to the nature of the transformation, MST-based (including NCST-based and NSST-based) fusion methods may not express and extract certain significant structures of source images properly without being sensitive to misregistration.

To address the misregistration problem in MST-based methods, sparse representation (SR) has emerged as another popular and powerful theory in the medical image fusion field (Liu and Wang, 2014; Liu et al., 2016, 2019; Fei et al., 2017). A typical SR-based medical image fusion method includes three basic steps: (1) a given dictionary is used to find the sparsest representation of source images; (2) some fusion rules are used to integrate the sparse representation coefficients; and (3) the integrated sparse representation coefficients and given dictionary are utilized to construct the fused image. For example, Liu and Wang (2014) proposed a novel adaptive sparse representation model for medical image fusion, where a set of more compact sub-dictionaries was learned to replace the single redundant dictionary in the traditional SR approach and achieved better results. Although the SR-based and extended methods are robust in terms of noise and misregistration to some extent, they cannot capture global information and suffer from significant energy loss.

In the field of medical image fusion, a key issue is to calculate a weight map since it reflects pixel activity information from different modality images, determining the quality of the final fused image. The weight map is calculated by two steps: activity level measurement and weight assignment. However, these two steps suffer from the robustness problem because traditional methods cannot deal with noise and misregistration well, as indicated in Liu et al. (2017). To improve the robustness of activity level measurement and weight assignment, Liu et al. (2017) introduced a deep learning fusion method with a simple multi-layer convolutional neural network (CNN) using the decision map and the medical image under the pyramid-based image fusion framework to reconstruct the fused medical image. While such a method achieves some success in specific medical image fusion tasks, this work may fail in multi-modal image fusion because the simple use of the CNN cannot extract fine-grained details efficiently.

To address the aforementioned challenges, we propose a novel hybrid medical image fusion framework with two principal elements (e.g., LatLRR and FCNs), inspired by Liu and Wang (2014) and Liu et al. (2017). The main contributions of this paper are as follows:

- The latent low-rank representation (LatLRR) is applied to decompose the medical image into low-rank (for extraction of details) and saliency components (for the preservation of energies).
- In the context of the low-rank component, to avoid the fixed-length feature vector from the final full connection layer and the information loss in the traditional CNN, three different FCNs (due to the nature of an input image of arbitrary size) are applied to produce a correspondingly-sized feature map with an efficient deconvolution layer (Guo et al., 2018), where a prediction is generated for each pixel and the spatial information in the original input image is retained. A sum strategy is used to fuse the saliency parts for energy preservation.
- To the best of our knowledge, this fusion strategy in combination with LatLRR and FCNs is the first to be applied in the medical image domain.

The remainder of this paper is structured as follows. In section 2, the proposed fusion strategy is described in detail. Section 3 gives the experimental configurations. Section 4 illustrates a comparative study between the proposed frameworks and five representative medical image fusion methods in terms of visual quality and quantitative and computational cost assessments. The conclusion is drawn in section 5.

## 2. Methodology

**Figure 1:**
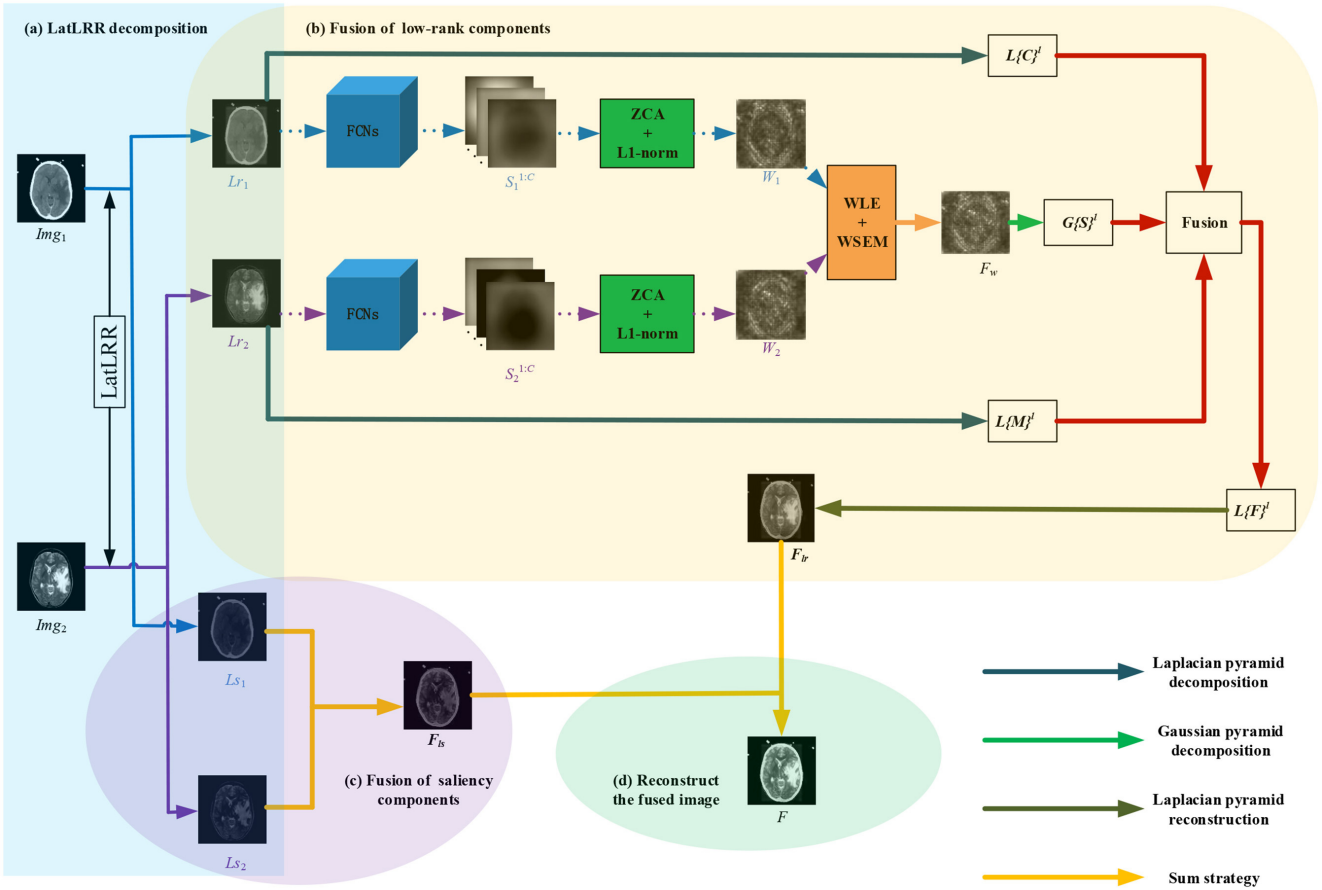
Schematic diagram of the proposed end-to-end frameworks (LatLRR-FCNs). The proposed LatLRR-FCNs enable the fusion image to extract details and preserve energies from paired sources. It is composed of four parts: **(a)** LatLRR decomposition, **(b)** fusion of low-rank components, **(c)** fusion of saliency components, and **(d)** reconstruction of fused image. *Img*_1_ and *Img*_2_ are the source medical images, *Lr*_1_ and *Lr*_2_ are the low-rank components of *Img*_1_ and *Img*_2_, *Ls*_1_ and *Ls*_2_ are the saliency components of *Img*_1_ and *Img*_2_, ${S_{1}}^{1:C}$ and ${S_{2}}^{1:C}$ are the score maps, *W*_1_ and *W*_1_ are the initial weight maps of *Lr*_1_ and *Lr*_2_, and the final fused weight map is *F*_*w*_. *F*_*lr*_ is the fused low-rank component, *F*_*ls*_ is the fused saliency component, and the final fused image is *F*.

As shown in Figure 1, each proposed framework is fed with a pair of pre-registered multi-modality medical source images, and outputs the fused medical image via the following four steps:

- We use the **LatLRR** theory to decompose the two medical source images into low-rank and saliency components (see section 2.1).
- To capture the detailed information of each source, a novel **fusion framework of the low-rank components** for each paired source based on FCNs, score maps, weight maps, and pyramid fusion is described (see section 2.2).
- To retain the energies of each source, a simple sum strategy is used to **fuse the saliency components** and **reconstruct the fused image** (see section 2.3).

### 2.1. LatLRR Decomposition

**Figure 2:**
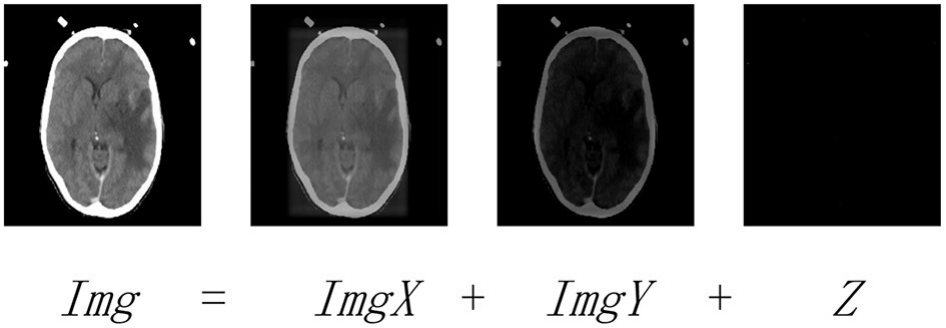
The LatLRR decomposing operation. *Img* is the observed image. *ImgX* and *ImgY* are the low-rank and saliency components of *Img*, respectively. *Z* denotes the noisy component.

The LatLRR theory was first proposed by Liu and Yan (2011), integrating subspace segmentation and feature extraction simultaneously, to extract the global and local structure from raw data in the context of natural images. It can be summarized into the following problem (Li and Wu, 2018):

(1) $$\begin{array}{l} \underset{X,Y,Z}{\min}{\left\|X\right\|}_{*}+{\left\|Y\right\|}_{*}+\lambda {\left\|Z\right\|}_{1}\\ s.t.Img=ImgX+ImgY+Z \end{array}$$

where ‖‖_*_ denotes the nuclear norm, ‖‖_1_ denotes the *l*_1_-norm, and λ > 0 is the balance coefficient. *Img* is the observed data matrix, and *X* and *Y* denote the low-rank and saliency coefficients, respectively. Note that Figure 2 explains the subject to Equation (1), where *ImgX*, *ImgY*, and *Z* are the low-rank, saliency, and noise components of *Img*, respectively.

In this paper, **the LatLRR decomposition** of Equation (1) can be solved by the inexact augmented Lagrangian multiplier method (Wang et al., 2013), where it extracts the low-rank and saliency components (e.g., *ImgX*_*j*_ and *ImgY*_*j*_) from medical image *Img*_*j*_ with *j* = 1, 2 (here, we consider two medical images, as shown in Figure 1a).

### 2.2. Fusion of Low-Rank Components

The **fusion of low-rank component** details can be seen in Figure 1b, including the FCN model for producing score maps (section 2.2.1), zero-phase component analysis (ZCA) (Kessy et al., 2018), and *l*_1_-norm operations (section 2.2.2) for whiting the score maps and generating the weight maps, respectively, weighted local energy (WLE) and weighted sum of eight-neighborhood-based modified Laplacian (WSEML) (Yin et al., 2018) operations (section 2.2.3) for obtaining the fused weight map, and pyramid fusion strategy (section 2.2.4) for reconstructing the fused low-rank component.

#### 2.2.1. FCN Model

**Figure 3:**
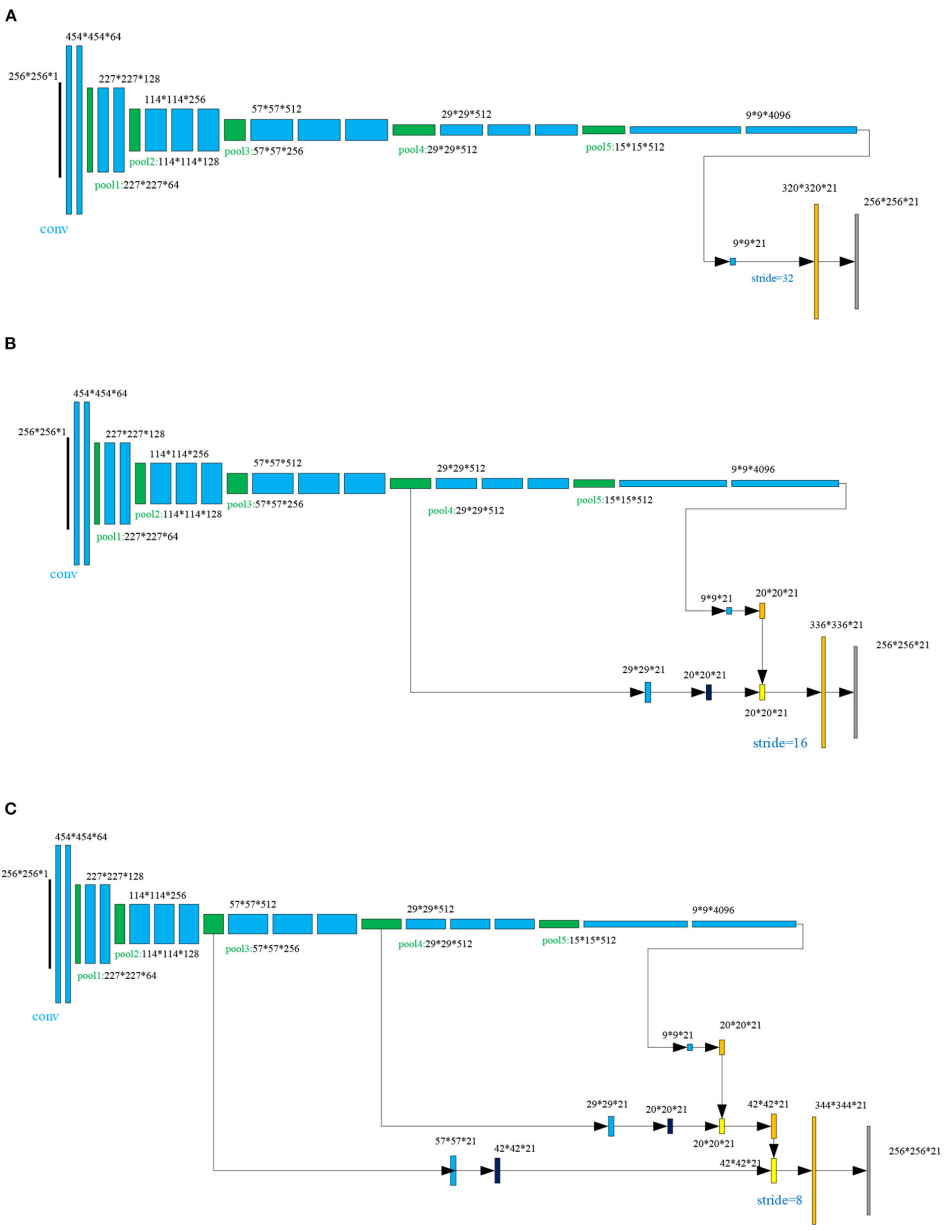
The skip architecture for upsampling for three scenarios: **(A)** FNC-32s, **(B)** FNC-16s, and **(C)** FNC-8s.

The fully convolutional networks (FCNs), demonstrated in many studies (Long et al., 2015; Wang L. et al., 2015; Chen et al., 2017; Guo et al., 2018), achieved significant performance in image semantic segmentation. In the FCN architecture, after multiple convolutions and pooling processes, the obtained image size will be progressively smaller with a lower resolution, resulting in a heatmap (coarse output). To keep the output the same size as the input, a skip architecture is used for upsampling. In this work, three different scenarios are tested, as shown in Figure 3. For each scenario, there are 38 layers of FCNs before upsampling, including 16 convolutional layers (blue color block in Figure 3A), 15 rule layers, five pooling layers (green color block in Figure 3A), and two dropout layers. In Figure 3A, the FCN-32s is a single-stream net in which up-samples stride 32 predictions back to pixels in a single step, but the upsampling output is very coarse. To obtain the refined outputs of FCN-16s, the final layer and the pool4 layer are used to combine the predictions in Figure 3B at stride 16. In Figure 3C, to obtain the outputs of FCN-8s with greater precision, the pool3 layer, the pool4 layer, and the final layer are utilized to combine the predictions at stride 8. As shown in Figure 1b, the three trained FCNs (FCN-32s, FCN-16s, and FCN-8s) are utilized to classify a pair of the low-rank components of medical source images *Lr*_1_ = *ImgX*_1_ and *Lr*_2_ = *ImgX*_2_ pixel by pixel, producing the corresponding score maps ${S_{j}}^{1:C},C=21,j=1,2$ (the choice of *C* = 21 can be seen in section 3.4).

#### 2.2.2. ZCA and *l*_1_-Norm Operations

**Figure 4:**
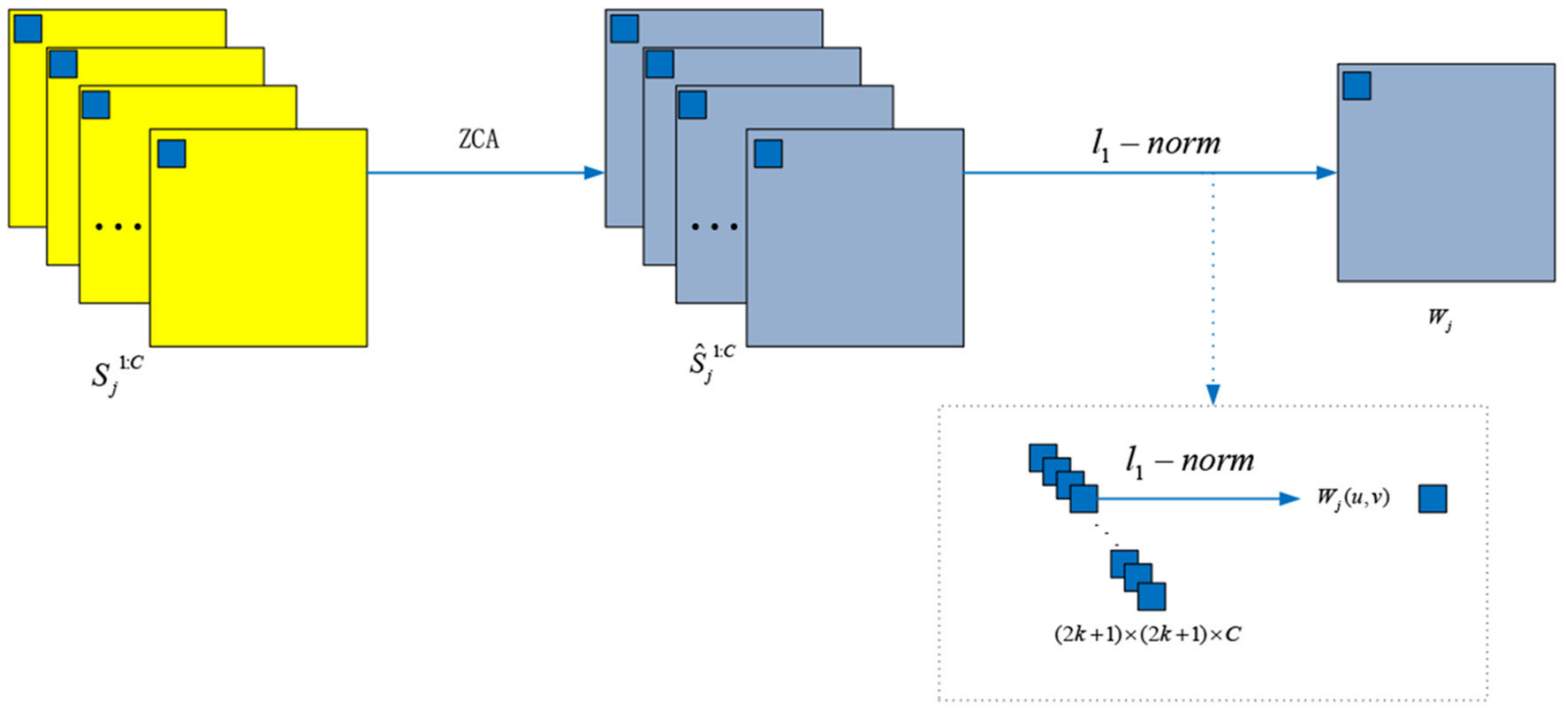
The ZCA and *l*_1_-norm operations for whiting the score maps and generating the weight maps, respectively.

The details for ZCA and *l*_1_-norm operations are depicted in Figure 4. To project the original redundancy score maps into a sparse subspace, we used ZCA to whiten those score maps ${S_{j}}^{1:C}$ and to obtain the score maps ${{Ŝ}_{j}}^{1:C}$. Among the ZCA, the covariance matrix $C{o_{j}}^{i}$ is decomposed as follows:

(2) $$\begin{array}{r} C{o_{j}}^{i}={S_{j}}^{i}\times {\left({S_{j}}^{i}\right)}^{T},\;C{o_{j}}^{i}=U\Sigma V^{T} \end{array}$$

where *i* = 1, 2, ⋯ , *C*; *j* = 1, 2, and *i* denote the *i*−*th* channel score map. Note that *U*, Σ and *V* define the left singular, singular values, and right singular matrixes, respectively (Chen et al., 2018). An alternative solution named ${{Ŝ}_{j}}^{i}$ is given as follows:

(3) $$\begin{array}{r} {{Ŝ}_{j}}^{i}={K_{j}}^{i}\times {S_{j}}^{i},\;{K_{j}}^{i}=U{\left(\Sigma +\eta I\right)}^{-\frac{1}{2}}U^{T} \end{array}$$

where η is a small value avoiding bad matrix inversion and *I* is the identity matrix. Then, the local *l*_1_-norm and average operations are used to calculate the initial weight map *W*_*j*_:

(4) $$\begin{array}{r} W_{j}=\frac{\sum_{x=u-k}^{u+k}\sum_{y=v-k}^{v+k}{\left||{{Ŝ}_{j}}^{i}\left(x,y\right)|\right|}_{1}}{\left(2k+1\right)\times \left(2k+1\right)} \end{array}$$

where *k* = 2 and the average *l*_1_-norm is calculated by a window centered at ${{Ŝ}_{j}}^{i}\left(u,v\right)$.

#### 2.2.3. WLE and WSEML Operations

Once the initial weight maps *W*_1_ and *W*_2_ is calculated, the WLE and WSEML are applied to acquire the final fused weight map *F*_*w*_, which is described in Figure 1b with the orange block.

First, the WLE of each *W*_*j*_ (i.e., Φ_*j*_) is calculated as follows:

(5) $$\begin{array}{r} \begin{array}{c} \Phi_{j}\left(u,v\right)=\overset{r}{\underset{p=-r}{\sum }}\overset{r}{\underset{q=-r}{\sum }}\left\{\Omega \left(p+r+1,q+r+1\right)W_{j}{\left(u+p,v+q\right)}^{2}\right\} \end{array} \end{array}$$

where *j* ∈ {1, 2} and Ω denote a (2*r*+1) × (2*r*+1) weighting matrix. The value of each element in Ω is 2^2*r*−*d*^ with radius *r*, *d* denotes the element of a four-neighborhood distance to the center. If *r* is 1, Ω is equal to

$$\frac{\text{1}}{\text{16}}\left[\begin{array}{ccc} \text{1} & \text{2} & \text{1}\\ \text{2} & \text{4} & \text{2}\\ \text{1} & \text{2} & \text{1} \end{array}\right]$$

Second, the WSEML of each *W*_*j*_ (i.e., Ψ_*j*_) is given as follows:

(6) $$\begin{array}{l} \Psi_{j}(u,v)=\sum_{p=-r}^{r}\sum_{q=-r}^{r}\{\Omega (p+r+1,q+r+1)\\ \;\times EML_{j}(u+p,v+q)\} \end{array}$$

where *EML* is expressed as follows:

(7) $$\begin{array}{r} \begin{array}{c} EML_{j}\left(u,v\right)=|2W_{j}\left(u,v\right)-W_{j}\left(u-1,v\right)-W_{j}\left(u+1,v\right)|\\ +|2W_{j}\left(u,v\right)-W_{j}\left(u,v-1\right)-W_{j}\left(u,v+1\right)|\\ +\frac{1}{\sqrt{2}}|2W_{j}\left(u,v\right)-W_{j}\left(u-1,v-1\right)-W_{j}\left(u+1,v+1\right)|\\ +\frac{1}{\sqrt{2}}|2W_{j}\left(u,v\right)-W_{j}\left(u-1,v+1\right)-W_{j}\left(u+1,v-1\right)| \end{array} \end{array}$$

Finally, the fused weight map *F*_*w*_ is calculated by the following rule:

(8) $$\begin{array}{r} F_{w}\left(u,v\right)=\left\{ \begin{array}{lll} W_{1}\left(u,v\right), & \text{if}\;\Phi_{1}\left(u,v\right)\cdot {\text{Ψ}}_{1}\left(u,v\right) & \geq \Phi_{2}\left(u,v\right)\cdot {\text{Ψ}}_{2}\left(u,v\right)\\ W_{2}\left(u,v\right), & \text{otherwise} \end{array}\right. \end{array}$$

#### 2.2.4. Pyramid Fusion Strategy

As shown in Figure 1b, the fused weight map *F*_*w*_ is decomposed into a Gaussian pyramid *G*{*S*}^*l*^ (green color arrow). The low-rank components *Lr*_1_ and *Lr*_2_ are decomposed into a Laplacian pyramid (dark blue color arrow) *L*{*C*}^*l*^ and *L*{*M*}^*l*^, respectively. Note that *l* denotes the *l*-th decomposition level, which is calculated by the following:

(9) $$\begin{array}{r} l=\lfloor \log_{2}\left(X,Y\right)\rfloor \end{array}$$

where ⌊·⌋ is the flooring operation and the spatial size of the low-rank component is *X* × *Y*.

Next, those coefficients about *L*{*F*} are calculated at each decomposition level *l*:

(10) $$\begin{array}{l} L{\left\{F\right\}}^{l}(u,\;v)\\ =\{\begin{array}{l} \left\{G{\left\{S\right\}}^{l}(u,\;v)\cdot L{\left\{C\right\}}^{l}(u,\;v\right)\\ +(1-G{\left\{S\right\}}^{l}(u,v))L{\left\{M\right\}}^{l}(u,\;v)\},\;if\{Q^{l}(u,\;v)\geq \tau \}\\ L{\left\{C\right\}}^{l}(u,\;v),\;if\{Q^{l}(u,\;v)<\tau \;\&\;E_{C}^{l}(u,\;v)\geq E_{M}^{l}(u,\;v)\}\\ L{\left\{M\right\}}^{l}(u,\;v),\;if\{Q^{l}(u,\;v)<\tau \;\&\;E_{C}^{l}(u,\;v)\geq E_{M}^{l}(u,\;v)\} \end{array} \end{array}$$

where the threshold τ determines the corresponding fusion mode. *Q*^*l*^(*x, y*) is given as follows:

(11) $$\begin{array}{r} Q^{l}\left(u,v\right)=\frac{2\underset{p}{\sum }\underset{q}{\sum }L{\left\{C\right\}}^{l}\left(u+p,v+q\right)L{\left\{M\right\}}^{l}\left(u+p,v+q\right)}{E_{C}^{l}\left(u,v\right)+E_{M}^{l}\left(u,v\right)} \end{array}$$

where $E_{C}^{l}\left(u,v\right)$ and $E_{M}^{l}\left(u,v\right)$ are the local energy maps of *L*{*C*}^*l*^ and *L*{*M*}^*l*^, respectively. $E_{C}^{l}\left(u,v\right)$ and $E_{M}^{l}\left(u,v\right)$ are defined as follows:

(12) $$\begin{array}{r} \begin{array}{c} E_{C}^{l}\left(u,v\right)=\underset{p}{\sum }\underset{q}{\sum }L{\left\{C\right\}}^{l}{\left(u+p,v+q\right)}^{2}\\ E_{M}^{l}\left(u,v\right)=\underset{p}{\sum }\underset{q}{\sum }L{\left\{M\right\}}^{l}{\left(u+p,v+q\right)}^{2} \end{array} \end{array}$$

Finally, the Laplacian pyramid reconstruction method (Mertens et al., 2009) (bottle green color arrow in Figure 1b) is used to reconstruct the fused low-rank components *F*_*lr*_ from *L*{*F*}^*l*^, as indicated in Equation (10).

### 2.3. The Flowchart of the Proposed LatLRR-FCNs

The FCN architectures (FCN-32s or FCN-16s or FCN-8s) are inserted to produce two score maps with the focus property after the LatLRR decomposition once a pair of low-rank components for two images are calculated (hereafter, we named the proposed LatRR-FCNs: including proposed LatRR-FCN-32s, LatRR-FCN-16s and LatRR-FCN-8s, respectively). Algorithm 1 provides a pseudo-code of the proposed LatRR-FCN-32s, LatRR-FCN-16s and LatRR-FCN-8s networks. Then, ZCA and *l*_1_-norm are utilized to white the score maps and obtain the initial weight maps for the low-rank components of paired source images [see **Part 2-(ii)** in Algorithm 1]. The WLE and WSEML techniques are used to fuse the two initial weight maps [see **Part 2-(iii)**]. The fused weight map and a pair of low-rank components under the pyramid-based image fusion framework (Mertens et al., 2009) are used to reconstruct the fused low-rank components' image *F*_*lr*_ [see **Part 2-(iv)**]. We sum the saliency components to obtain the fused saliency components' image *F*_*ls*_ (see **Part 3**). Finally, the fused image *F* is obtained by combining *F*_*lr*_ and *F*_*ls*_ (see **Part 4**).

**Algorithm 1:** LatRR-FCN-32s, LatRR-FCN-16s, and LatRR-FCN-8s networks.

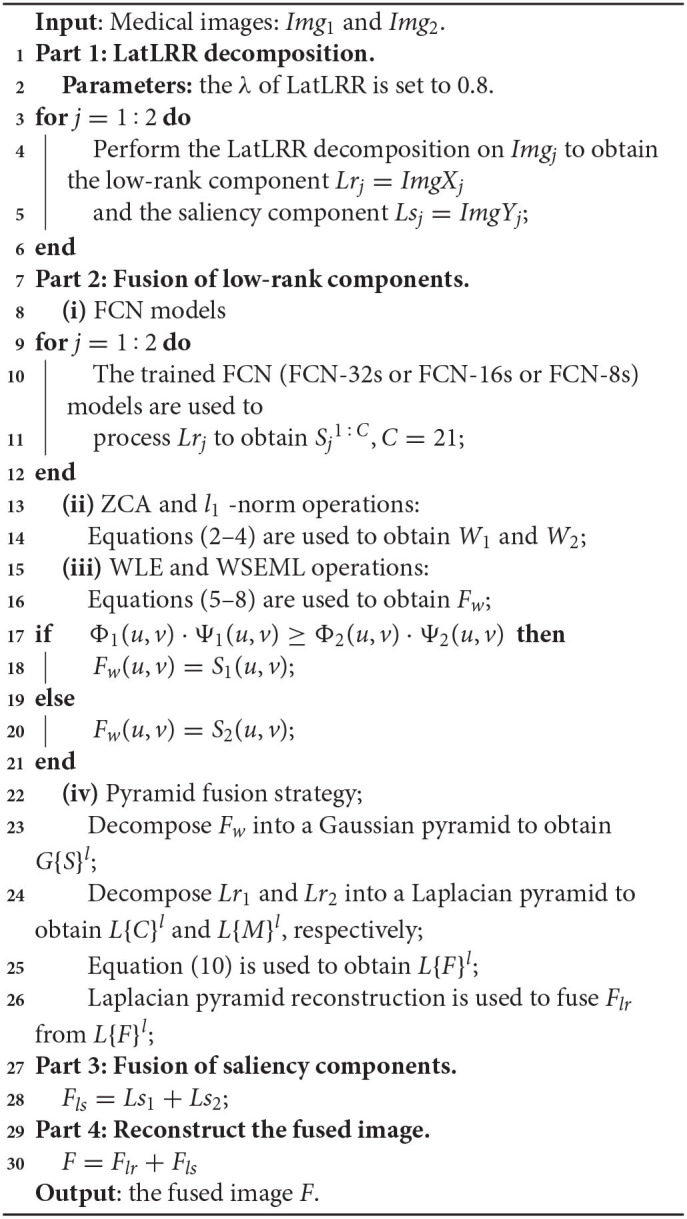

## 3. Experimental Configurations

### 3.1. FCN Training Sets

Currently, transfer learning (Bar et al., 2015; Liu et al., 2017; Razzak et al., 2018; Lu et al., 2019, 2020) has become an active topic in the field of medical image analysis. In this study, we directly adopted a transfer learning strategy, and we trained the FCNs (FCN-32s, FCN-16s, and FCN-8s) on the PASCAL VOC 2012 dataset (Everingham et al., 2012) and the semantic boundary dataset (SBD) (Hariharan et al., 2011). The PASCAL VOC 2012 dataset contains 20 foreground object classes and 1 background class. The original dataset contains 1,464 (train), 1,449 (val), and 1,456 (test) pixel-level annotated images. The dataset is augmented with the SBD by extra annotations (Mertens et al., 2009), resulting in 10,582 training images.

### 3.2. Source Medical Image Testing Sets

In our experiments, we used 40 pairs of multi-modal medical images (each medical image fusion problem contains 10 image pairs) to demonstrate the usefulness and efficiency of the proposed methods. Most of the test images were gathered from the Whole Brain Atlas databases (Vidoni, 2012) and have been widely adopted in previous related publications (Liu and Wang, 2014; Liu et al., 2017, 2019; Yin et al., 2018; Zhu et al., 2019). Each pair of images was geometrically aligned, and all the test images were normalized to 256 × 256.

### 3.3. State-of-the-Art Methods

Five superior medical image fusion methods were collected for comparison against our proposed methods. These included the adaptive sparse representation (ASR) method (Liu and Wang, 2014) (https://github.com/yuliu316316/MST-SR-Fusion-Toolbox), the convolutional neural network (CNN)-based (LP-CNN) method (Liu et al., 2017) (https://github.com/yuliu316316/CNN-Fusion), the phase congruency and local Laplacian energy-based NSCT (NSCT-PC-LLE) method (Zhu et al., 2019) (https://github.com/zhiqinzhu123/Source-code-of-medical-image-fusion-in-NSCT-domain), the parameter-adaptive pulse coupled-neural network (NSST-PAPCNN) in the NSST domain method (Yin et al., 2018) (https://github.com/yuliu316316/NSST-PAPCNN-Fusion), and the convolutional sparsity-based morphological component analysis (CSMCA) method (Liu et al., 2019) (https://github.com/yuliu316316/CSMCA-Fusion). Among them, the NSCT-PC-LLE, NSST-PAPCNN, and CSMCA methods were proposed in last year.

### 3.4. Parameter Choices

**Figure 5:**
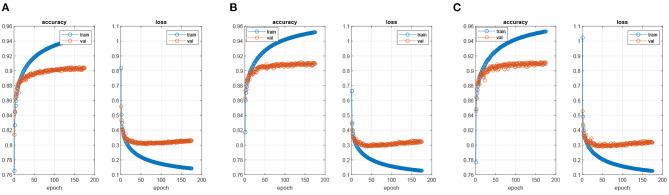
The process of training FCN models. **(A)** FNC-32s model, **(B)** FNC-16s model, and **(C)** FNC-8s model.

The parameters of all compared methods were set to the default values. The key parameters for our proposed algorithms were given in Table 1. According to this table, the parameter λ in LatLRR decomposition was 0.8 (Li and Wu, 2018), and the threshold τ in Equation (10) was set to 0.8 (Liu et al., 2017). The PASCAL VOC 2012 dataset contained 20 foreground object classes and one background class, so that the *C* in ${S_{j}}^{1:C}$ was equal to 21. Note that we adopted a transfer learning strategy directly to train the FCN-VGG16 (Long et al., 2015) by MacInnes, and the trained models were obtained after 50 epochs using the training data. The choice of epoch was dependent on Figure 5. When the epoch was lower than 50, the accuracy of the training and validation sets increased with the values of epochs. However, the accuracy of the validation set leveled off when the epoch was higher than 50, although the accuracy of the training set still increased regardless of the scenario (FCN-32s, FCN-16s, and FCN-8s). In terms of the loss function, the values for all FCN architectures decreased with the epoch in the case of the training set, but the loss of the scenarios tended to converge at the 50 epochs. Therefore, to balance the computational complexity and accuracy, the epochs for FCN models in this paper were chosen as 50.

**Table 1:** The key parameters used in our algorithms.

**LatLRR decomposition**	**Threshold**	**Classes**	**No. of epochs**
**λ**	**τ**	**C**	***epoch***
0.8	0.8	21	50

### 3.5. Experimental Environment

All the experiments were implemented in MATLAB R2019a on a WIN64 Intel(R) Core (TM)i7-8750H CPU@2.20GHz 8GB RAM. The training models of the proposed method were trained in MATLAB R2019a+VS2017+ MatConvNet 1.0-beta25.

### 3.6. Objective Evaluation Metrices

In this study, five common representative quantitative metrics, e.g., *EN* (Liu and Wang, 2014), *Q*_*MI*_ (Bhatnagar et al., 2013), *Q*^*AB*/*F*^ (Xydeas et al., 2000), *SCD* (Aslantas and Bendes, 2015), and *VIFF*(Han et al., 2013) (for all metrics, a larger value indicates a better performance), were used to evaluate the quality of fused images. The metrics were briefly described as follows:

**(i) Entropy (EN)** Liu and Wang (2014), Wang et al. (2017), Zhang et al. (2017): Entropy measures the amount of information in the fused image.

**(ii) Mutual information (MI) of two images *Q*_*MI*_** Bhatnagar et al. (2013): MI is a quantitative assessment of the information shared by two images. Mathematically, MI can be expressed with joint entropy *H*(*C, D*), marginal entropy *H*(*C*), and *H*(*D*) of two variables *C* and *D* as follows:

(13) $$\begin{array}{r} MI\left(C,D\right)=H\left(C\right)+H\left(D\right)-H\left(C,D\right) \end{array}$$

where $H\left(C\right)=-\underset{c}{\sum }p\left(c\right)\log_{2}p\left(c\right),H\left(D\right)=-\underset{d}{\sum }p\left(d\right)\log_{2}p\left(d\right),H\left(C,D\right)=-\underset{c,d}{\sum }p\left(c,d\right)\log_{2}p\left(c,d\right)$. *p*(*c*) and *p*(*d*) denote the marginal probability distributions of *C* and *D*, respectively. *p*(*c, d*) denotes the joint probability distribution of *C* and *D*. Therefore, the quality of the fused image with respect to input images *Img*_1_ and *Img*_2_ can be defined as:

(14) $$\begin{array}{r} Q_{MI}=2\left[\frac{MI\left(Img_{1},F\right)}{H\left(Img_{1}\right)+H\left(F\right)}+\frac{MI\left(Img_{2},F\right)}{H\left(Img_{2}\right)+H\left(F\right)}\right] \end{array}$$

**(iii) Edge-based similarity measure *Q*^*AB*/*F*^**: The authors in Xydeas et al. (2000) proposed a metric *Q*^*AB*/*F*^ to produce the similarity between the edges that transform in the fusion process. This metric is defined as follows:

(15) $$\begin{array}{r} Q^{AB/F}=\frac{\overset{N}{\underset{u=1}{\sum }}\overset{M}{\underset{v=1}{\sum }}\left(Q^{AF}\left(u,v\right)w^{A}\left(u,v\right)+Q^{BF}\left(u,v\right)w^{B}\left(u,v\right)\right)}{\overset{N}{\underset{u=1}{\sum }}\overset{M}{\underset{v=1}{\sum }}\left(w^{A}\left(u,v\right)+w^{B}\left(u,v\right)\right)} \end{array}$$

where *A, B*, and *F* represent the two input images (*Img*_1_ and *Img*_2_) and fused images. The size of each image is *N* × *M*, *Q*^*AF*^(*u, v*) and *Q*^*BF*^(*u, v*) are defined as follows:

(16) $$\begin{array}{r} \begin{array}{c} Q^{AF}\left(u,v\right)=Q_{g}^{AF}\left(u,v\right)Q_{\alpha }^{AF}\left(u,v\right)\\ Q^{BF}\left(u,v\right)=Q_{g}^{BF}\left(u,v\right)Q_{\alpha }^{BF}\left(u,v\right) \end{array} \end{array}$$

where $Q_{g}^{*F}\left(u,v\right)$ and $Q_{\alpha }^{*F}\left(u,v\right)$ are the edge strength and orient preservation values at location (*u, v*) in images *A* and *B*, respectively. The dynamic range for *Q*^*AB*/*F*^ is equal to [0, 1], where a larger value for *Q*^*AB*/*F*^ indicates a better fusion result. For more details of this metric, please refer to Xydeas et al. (2000).

**(iv) The sum of the correlations of differences (SCD)** Aslantas and Bendes (2015) is a quality metric formulated as follows:

(17) $$\begin{array}{r} SCD=r\left(D_{1},Img_{1}\right)+r\left(D_{2},Img_{2}\right) \end{array}$$

where *D*_1_ = *F* − *Img*_2_, *D*_2_ = *F* − *Img*_1_, *F* is the fused image, and *Img*_1_ and *Img*_2_ are the input images. The *r*(.) function calculates the correlation between *S*_*k*_ and *D*_*k*_, given as:

(18) $$\begin{array}{r} r\left(D_{k},Img_{k}\right)\\ =\frac{\underset{u}{\sum }\underset{v}{\sum }\left(D_{k}\left(u,v\right)-{\bar{D}}_{k}\right)\left(Img_{k}\left(u,v\right)-{\bar{Img}}_{k}\right)}{\sqrt{\left(\underset{u}{\sum }\underset{v}{\sum }j{\left(D_{k}\left(u,v\right)-{\bar{D}}_{k}\right)}^{2}\right)\underset{u}{\sum }\underset{v}{\sum }{\left(Img_{k}\left(u,v\right)-{\bar{Img}}_{k}\right)}^{2}}} \end{array}$$

where *k* = 1, 2, ${\bar{D}}_{k}$ and ${\bar{Img}}_{k}$ are the average of the pixel values of *D*_*k*_ and *Img*_*k*_, respectively.

**(v) The human visual perception-based metric visual information fidelity fusion (VIFF)** Han et al. (2013): To obtain the *VIFF*, four steps are needed. First, the source and fused images are filtered and then divided into blocks. Second, visual information is evaluated with and without distortion information in each block. Third, the *VIFF* of each sub-band is calculated. Finally, the overall quality measure is determined by weighting the *VIFF* of each sub-band.

### 3.7. Color Space Fusion

In our proposed methods, the YUV color space was used to solve the grayscale and RGB color image (PET, SPECT) fusion issues. First, the RGB color image was converted into a YUV color space, resulting in three channel components of Y, U, and V. Then, the grayscale image and the Y channel were fused by using the proposed fusion methods, as described in section 2. Finally, the fused Y-channel component, the U-channel component, and the V-channel component were inversely transformed by YUV space, obtaining the fused color image.

## 4. Results and Discussion

This section is devoted to showing that the proposed LatRR-FCNs can improve the information details and energy preservation in terms of **visual quality assessment** (section 4.1), **quantitative assessment** (section 4.2) and **computational cost assessment** (section 4.3), compared with five recently proposed methods: ASR (Liu and Wang, 2014), LP-CNN (Liu et al., 2017), NSCT-PC-LLE (Zhu et al., 2019), NSST-PAPCNN (Yin et al., 2018), and CSMCA (Liu et al., 2019). In this study, the usefulness and efficiency of each method are investigated with four sets of medical image fusion studies, including CT and MR, MR-T1 and MR-T2, PET and MR, and SPECT and MR.

### 4.1. Visual Quality

**Figure 6:**
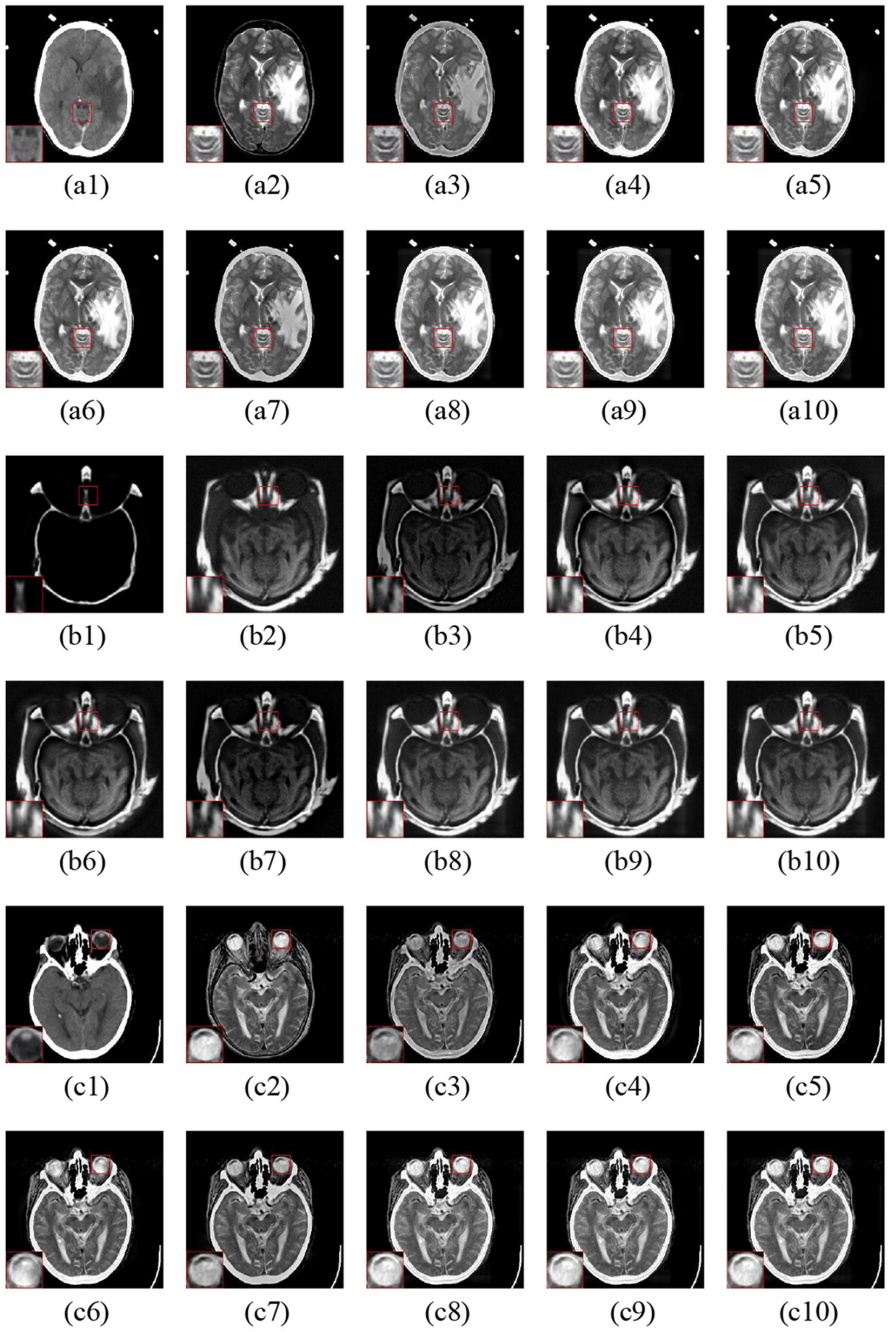
Three fusion examples of CT and MR images. One close-up is provided in each set for better comparison. The original images: **(a1–c1)** CT and **(a2–c2)** MR. The fusion results **(a3–c3)** ASR, **(a4–c4)** NSCT-PC-LLE, **(a5–c5)** LP-CNN, **(a6–c6)** NSST-PAPCNN, **(a7–c7)** CSMCA, **(a8–c8)** LatLRR-FCN-32s, **(a9–c9)** LatLRR-FCN-16s, and **(a10–c10)** LatLRR-FCN-8s.

The fusion examples of CT and MR images are given in Figure 6. Furthermore, one representative region of each result is enlarged for better comparison. The ASR and CSMCA methods reveal a significant energy loss in both the CT and MR images (resulting in an intensity and contrast decrease in the fused images), especially for the bone and lesion regions in the Figures 6a3–c3,a7–c7. The fusion results of the NSCT-PC-LLE, LP-CNN, NSST-PAPCNN, and the proposed methods have better information preservation for the CT and MR modalities. However, the NSCT-PC-LLE, LP-CNN, and NSST-PAPCNN methods cannot extract the detailed information well in the MR image, which can be seen in the Figures 6a4–c4,a5–c5,a6–c6 and the corresponding highlighted close-ups. Furthermore, the ASR method fails to extract the structural and edge details from the CT modality (see Figures 6a4–c4). The NSCT-PC-LLE and NSST-PAPCNN methods outperform the ASR method, even though some structural details cannot be extracted (see the Figures 6a3–c3,a4–c4,a6–c6). The proposed frameworks and LP-CNN method can effectively extract the structure and edge details from both CT and MR modalities (see Figures 6a8–a10,b8–b10,c8–c10,a5–c5, respectively). The proposed methods perform well on the preservation of detailed and structural information for all three examples.

**Figure 7:**
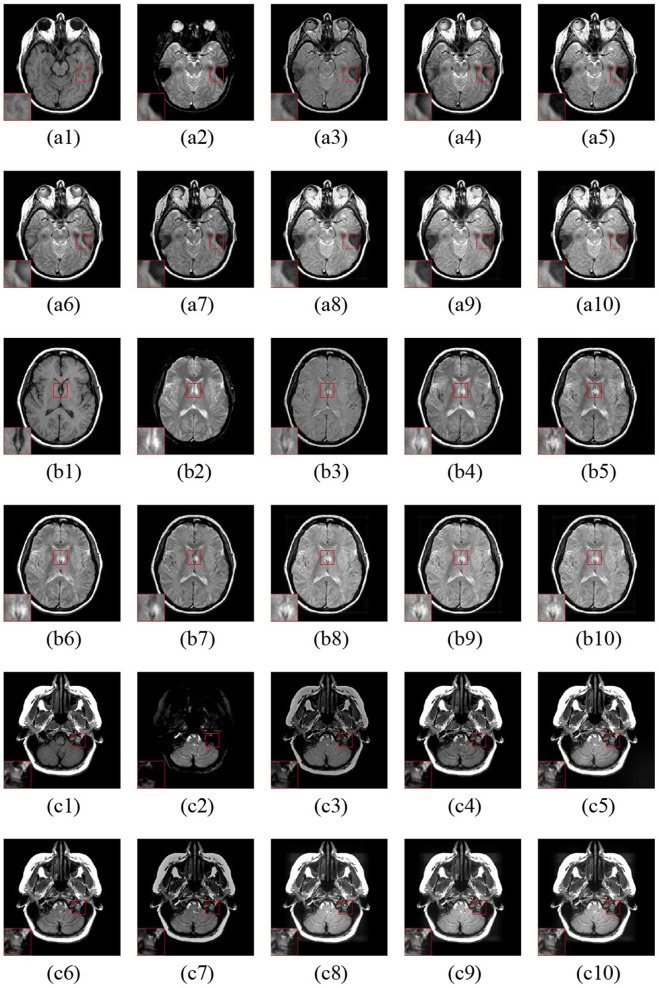
Three fusion examples of MR-T1 and MR-T2 images. One close-up is provided in each set for better comparison. The original images: **(a1–c1)** MR-T1 and **(a2–c2)** MR-T2. The fusion results are as follows: **(a3–c3)** ASR, **(a4–c4)** NSCT-PC-LLE, **(a5–c5)** LP-CNN, **(a6–c6)** NSST-PAPCNN, **(a7–c7)** CSMCA, **(A8–C8)** LatLRR-FCN-32s, **(a9–c9)** LatLRR-FCN-16s, and **(a10–c10)** LatLRR-FCN-8s.

Figure 7 gives three fusion examples of MR-T1 and MR-T2 images. The ASR and CSMCA methods suffer from low intensity and contrast caused by the loss of energy (see the Figures 7a3–c3,a7–c7 with the close-up). In addition, the NSCT-PC-LLE, LP-CNN, and NSST-PAPCNN methods cannot preserve the detailed information (see the close-ups in Figures 7a4–c4,a5–c5,a6–c6, respectively). Furthermore, the ASR and NSCT-PC-LLE methods exhibit lower ability in structure and edge detail extraction within the MR-T1 modality, explained by the close-up in Figures 7a4–c4,a5–c5. Finally, compared to the other tested methods, our proposed LatLRR-FCN-based methods achieve the best performance, as shown with the close-ups in Figures 7a8–c8,a9–c9,a10–c10, respectively.

**Figure 8:**
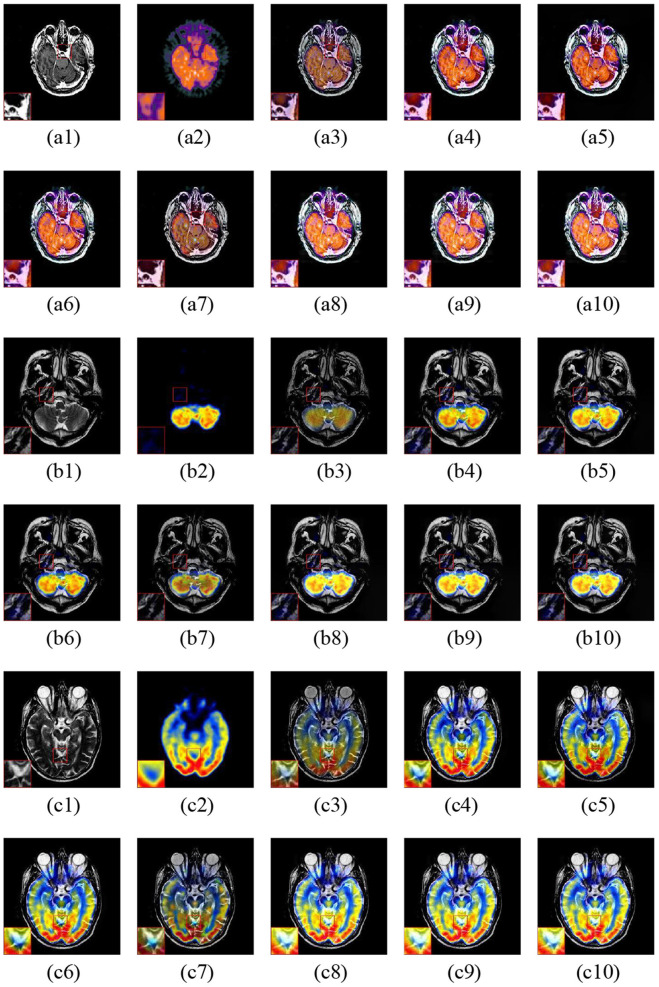
Three fusion examples of MR and PET images. One close-up is provided in each set for better comparison. The original images: **(a1–c1)** MR and **(a2–c2)** PET. The fusion results: **(a3–c3)** ASR, **(a4–c4)** NSCT-PC-LLE, **(a5–c5)** LP-CNN, **(a6–c6)** NSST-PAPCNN, **(a7–c7)** CSMCA, **(a8–c8)** LatLRR-FCN-32s, **(a9–c9)** LatLRR-FCN-16s, and **(a10–c10)** LatLRR-FCN-8s.

Figure 8 shows the three fusion examples of MR and PET images. The ASR and CSMCA methods lose a significant amount of energy in both the MR and PET modalities, as viewed in the Figures 8a3–c3,a7–c7 and the corresponding close-ups. Note that the NSCT-PC-LLE and LP-CNN methods are subjected to a severe color distortion (see the close-ups in Figures 8a4–c4,a5–c5). Furthermore, the color distortion existed more or less in the fusion results of the NSST-PAPCNN method (see Figures 8a7–c7 and the close-ups). Overall, the color preservation of our proposed algorithms (see Figures 8a8–c8,a9–c9,a10–c10 together with their close-ups) are also significantly higher than the other methods.

**Figure 9:**
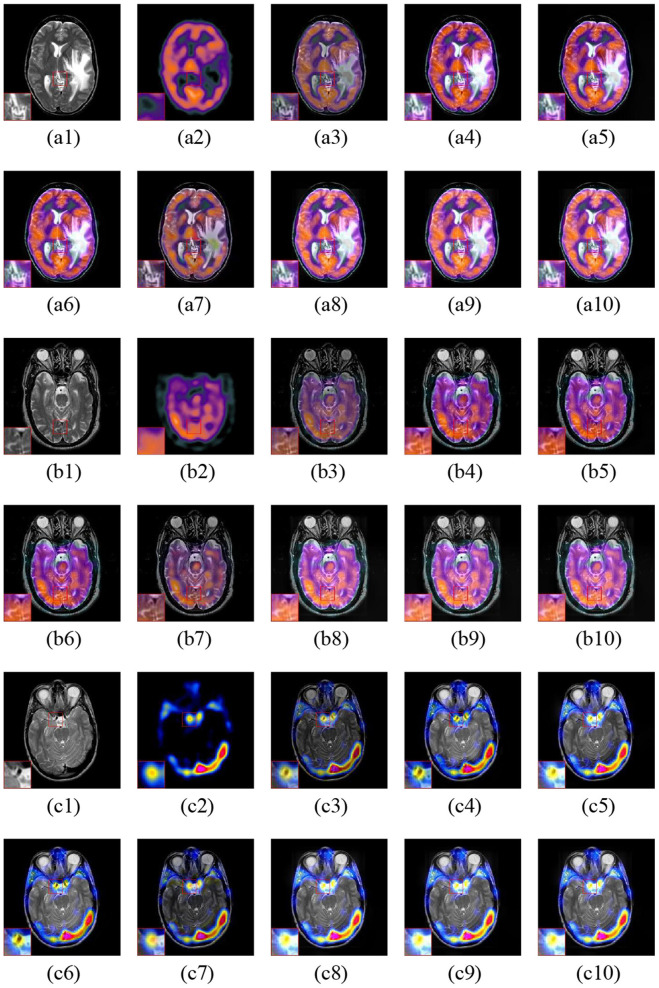
Three fusion examples of MR and SPECT images. One close-up is provided in each set for better comparison. The original images: **(a1–c1)** MR and **(a2–c2)** SPECT. The fusion results: **(a3–c3)** ASR, **(a4–c4)** NSCT-PC-LLE, **(a5–c5)** LP-CNN, **(a6–c6)** NSST-PAPCNN, **(a7–c7)** CSMCA, **(a8–c8)** LatLRR-FCN-32s, **(a9–c9)** LatLRR-FCN-16s, and **(a10–c10)** LatLRR-FCN-8s.

The fusion examples of three sets of MR and SPECT images are shown in Figure 9. The ASR and CSMCA methods still lose much energy in both the MR and PET modalities (see Figures 9a3–c3,a7–c7). Moreover, color distortion exists in the NSCT-PC-LLE and LP-CNN methods (see the close-up in Figures 9a4–c4,a5–c5). Furthermore, in the results of the NSST-PAPCNN method, color distortion also exists (in Figures 9a6–c6, especially the close-up). The visual quality of color preservation of our proposed methods significantly outperforms the others.

### 4.2. Quantitative Assessment

Here, five common quantitative metrics as described in section 3.6 are employed to appraise the fusion performance. The average score of each method in each fusion problem is reported in Table 2. The top three values of all the fusion methods are shown in bold, and their rank is indicated by a superscript. For CT and MRI fusion, the proposed methods achieve the best performance in terms of *EN* (i.e., the values of *EN* for LatLRR-FCN-8s, LatLRR-FCN-16s, and LatLRR-FCN-32s are equal to **5.4531^1^**, **5.4037^2^** and **5.3915^3^**, respectively), *Q*_*MI*_ (the value of *Q*_*MI*_ for LatLRR-FCN-32s, **1.2072^1^**, higher than that of LatLRR-FCN-16s, **1.2065^2^**, and LatLRR-FCN-8s, **1.2043^3^**), *SCD*, and *VIFF* metrics. Note that in the context of the *Q*^*AB*/*F*^ metric, our proposed LatLRR-FCN-32s (*Q*^*AB*/*F*^ = **0.5541^2^**) is slightly lower than the LP-CNN method (*Q*^*AB*/*F*^ = **0.5688^1^**) but slightly superior to the CSMCA method (*Q*^*AB*/*F*^ = **0.5535^3^**). In the case of MR-T1 and MR-T2 fusion, the proposed methods show the best values in three of the five metrics with *EN*, *SCD* and *VIFF*. Among them, an increase improvement in the proposed LatLRR-FCN-32s for *SCD* about **10.23%** [(1.7483 − 1.5694)/1.7483 = 0.1023] is reported in Table 2, compared to the best performance among the other five methods, i.e., NSST-PAPCNN algorithm. For MRI and PET fusion, overall, the proposed LatRR-FCNs obtain the best results in all five objective metrics except that the NSST-PAPCNN method achieves the rank first in the metric *Q*^*AB*/*F*^ (i.e., *Q*^*AB*/*F*^ = **1.0946^1^**) with a slight improvement **1.16%** (e.g., 0.0116 = (1.0946 − 1.0819)/1.0946) compared with our proposed LatLRR-FCN-8s. Finally, our proposed LatRR-FCNs outperform the other fusion methods in the aspect of *EN*, *SCD*, and *VIFF* metrics for MRI and SPECT fusion, especially for the *SCD* metric of LatRR-FCN-8s with a significant improvement in **17.22%** (0.1722 = (1.8265−1.5119)/1.8265) compared to that of the NSST-PAPCNN approach.

**Table 2:** Five metrics of different methods for the four medical image fusion problems (a higher value for each metric indicates a better performance).

**CT and MR**	***EN***	***Q*_*MI*_**	***Q*^*AB*/*F*^**	***SCD***	***VIFF***
ASR	4.7319	1.1068	0.5378	1.5400	0.3588
NSCT-PC-LLE	5.1619	1.1930	0.5206	1.5786	0.4570
LP-CNN	4.9503	1.1674	**0.5688^1^**	1.5715	0.4476
NSST-PAPCNN	5.2323	1.1687	0.5383	1.6259	0.4703
CSMCA	4.8532	1.1120	**0.5535^3^**	1.5462	0.4135
LatLRR-FCN-32s	**5.3915^3^**	**1.2072^1^**	**0.5541^2^**	**1.6946^3^**	**0.4998^3^**
LatLRR-FCN-16s	**5.4037^2^**	**1.2065^2^**	0.5531	**1.6986^2^**	**0.5003^2^**
LatLRR-FCN-8s	**5.4531^1^**	**1.2043^3^**	0.5517	**1.7034^1^**	**0.5007^1^**
**MR-T1 and MR-T2**	***EN***	***Q*_*MI*_**	***Q*^*AB*/*F*^**	***SCD***	***VIFF***
ASR	4.0307	0.9965	0.5916	1.4834	0.4553
NSCT-PC-LLE	4.5478	**1.1500^1^**	**0.6432^3^**	1.4883	0.5934
LP-CNN	4.6327	**1.1294^3^**	**0.6670^1^**	1.5333	0.6006
NSST-PAPCNN	4.6654	**1.1305^2^**	0.6035	1.5694	0.6053
CSMCA	4.1134	1.0401	**0.6606^2^**	1.4677	0.5421
LatLRR-FCN-32s	**5.0259^3^**	1.0940	0.6380	**1.7483^1^**	**0.6335^3^**
LatLRR-FCN-16s	**5.0524^2^**	1.0953	0.6384	**1.7475^2^**	**0.6346^2^**
LatLRR-FCN-8s	**5.0949^1^**	1.0974	0.6388	**1.7457^3^**	**0.6358^1^**
**PET and MR**	***EN***	***Q*_*MI*_**	***Q*^*AB*/*F*^**	***SCD***	***VIFF***
ASR	3.9649	0.9899	0.6153	1.6446	0.4206
NSCT-PC-LLE	4.4502	1.0739	0.6547	1.7013	0.5789
LP-CNN	4.5261	1.0523	0.6540	1.7121	0.5828
NSST-PAPCNN	4.4790	**1.0946^1^**	0.6486	1.7398	0.5997
CSMCA	4.0233	1.0078	0.6573	1.6725	0.5015
LatLRR-FCN-32s	**4.5648^1^**	1.0808	**0.6588^1^**	**1.8861^1^**	**0.6699^1^**
LatLRR-FCN-16s	**4.5556^3^**	**1.0816^3^**	**0.6587^2^**	**1.8851^2^**	**0.6693^2^**
LatLRR-FCN-8s	**4.5573^2^**	**1.0819^2^**	**0.6584^3^**	**1.8834^3^**	**0.6689^3^**
**SPECT and MR**	***EN***	***Q*_*MI*_**	***Q*^*AB*/*F*^**	***SCD***	***VIFF***
ASR	4.5089	1.1563	0.5548	1.3995	0.4692
NSCT-PC-LLE	4.9146	**1.3703^2^**	**0.6405^2^**	1.3839	0.5629
LP-CNN	5.4404	**1.3146^3^**	**0.6496^1^**	1.4958	0.6024
NSST-PAPCNN	4.9242	**1.3912^1^**	**0.6355^3^**	1.5119	0.5814
CSMCA	4.6187	1.1977	0.6233	1.4073	0.5339
LatLRR-FCN-32s	**5.6361^1^**	1.2880	0.6205	**1.8224^3^**	**0.7075^3^**
LatLRR-FCN-16s	**5.6270^3^**	1.2899	0.6207	**1.8244^2^**	**0.7078^2^**
LatLRR-FCN-8s	**5.6341^2^**	1.2916	0.6210	**1.8265^1^**	**0.7081^1^**

As also shown in Table 2, for different metrics, it can be concluded as follows. (1) For the *EN* metric, our proposed techniques have the optimal energy preservation in four medical image fusion problems. (2) The *Q*_*MI*_ metric shows that our proposed LatLRR-FCN-8s and LatLRR-FCN-16s architectures obtain the best performance in detail information extraction than others in the context of CT and MR image fusion and PET and MR image fusion problems. (3) In terms of the *Q*^*AB*/*F*^ metric, our proposed frameworks are also close to the other comparison algorithms in edge and direction retention. (4) For the *SCD* metric, our proposed methods have a higher cross-correlation between the fused image and the input image than the others in all four medical image fusion problems. (5) For the *VIFF* metric, compared to the other methods, our proposed approaches are more consistent with the visual mechanism of human eyes in four medical image fusion problems.

**Figure 10:**
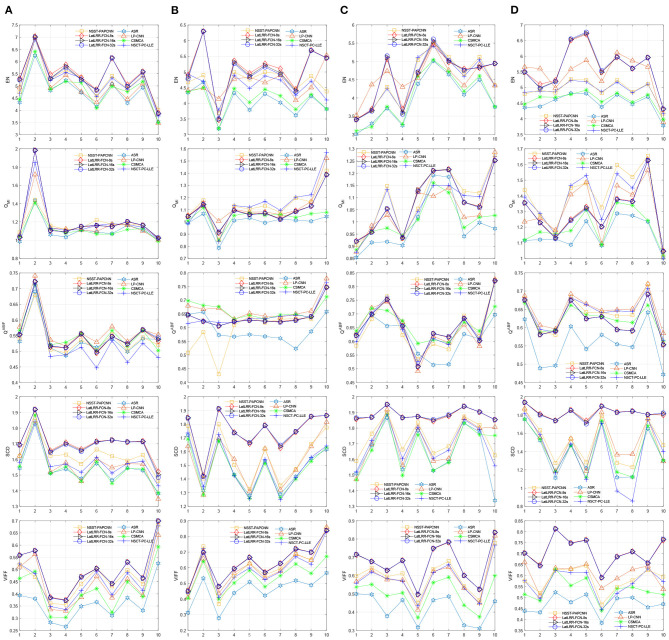
Objective performance of different fusion methods in each fusion problem. **(A)** CT and MR, **(B)** MR-T1 and MR-T2, **(C)** PET and MR, and **(D)** SPECT and MR.

Moreover, Figure 10 shows the objective performance of different methods in each fusion problem. The ten scores of each method in each fusion problem are connected for each metric. Obviously, the proposed three methods show the optimal performance among them. More specifically, the proposed LatLRR-FCNs are the best three ranks on the metrics of *EN*, *SCD*, and *VIFF* for all four problems, which is also concluded in Table 2.

### 4.3. Computational Cost Assessment

The average computational costs of different methods are shown in Table 3, including gray-level and color images. Although the performances of LP-CNN, NSCT-PC-LLE, and NSST-PAPCNN are better than the proposed methods, the proposed methods achieve a better performance in terms of both visual perception and objective assessment. However, the processing cost of ASR and CSMCA is 6 times and 10 times higher than our proposed methods. In total, the experimental results show that the proposed methods can achieve competitive performance in terms of computational costs in practice.

**Table 3:** Computation cost of different methods.

**Methods**	**Gray-level image fusion time (s)**	**Color image fusion time (s)**
ASR	128.2032	163.4961
NSCT-PC-LLE	2.1638	4.2115
LP-CNN	11.7130	11.9583
NSST-PAPCNN	4.2226	4.8991
CSMCA	233.4387	252.1574
LatLRR-FCN-32s	21.5983	19.5076
LatLRR-FCN-16s	21.7452	19.7250
LatLRR-FCN-8s	20.2011	18.7253

## 5. Conclusion

In this paper, three LatRR-FCNs have been proposed to improve energy conservation and detail extraction during medical image fusion. Based on LatLRR, the LatRR-FCNs decompose the medical image into low-rank and saliency components, which can enhance the extraction of detail in the SR-based methods. Then, three different fully convolutional networks (FCN-32s, FCN-16s, and FCN-8s), ZCA, *l*_1_-norm, WLE, and WSEML operations together with a pyramid-based fusion method are applied to fuse the low-rank components, which can simultaneously enhance the energy preservation and detail extraction. We sum the saliency components to obtain the fused saliency components. Finally, the fused image is obtained by combining the fused low-rank components and fused saliency components. The proposed frameworks were evaluated in the context of four kinds of medical image fusion problems, including CT and MR, MR-T1 and MR-T2, PET and MR, and SPECT and MR. The results of our experiments demonstrated that the proposed frameworks can achieve optimal performance in both visual quality and objective assessment.

## Data Availability Statement

Publicly available datasets were analyzed in this study. This data can be found here: http://www.med.harvard.edu/aanlib/.

## Author Contributions

ZX and JL: conceptualization. ZX and WX: methodology and writing–review and editing. ZX: software, visualization, and writing–original draft preparation. SZ, CM-C, and ZC: validation. RZ and XC: formal analysis. JL: resources and supervision. BL: data curation and project administration. All authors have read and agreed to the published version of the manuscript.

## Conflict of Interest

The authors declare that the research was conducted in the absence of any commercial or financial relationships that could be construed as a potential conflict of interest. The handling editor declared a past co-authorship with one of the authors XC.

## References

[B1] Anitha S. Subhashini T. Kamaraju M. (2015). A novel multimodal medical image fusion approach based on phase congruency and directive contrast in nsct domain. Int. J. Comput. Appl. 129, 30–35. 10.5120/ijca2015907014

[B2] Aslantas V. Bendes E. (2015). A new image quality metric for image fusion: the sum of the correlations of differences. AEU Int. J. Electron. Commun. 69, 1890–1896. 10.1016/j.aeue.2015.09.004

[B3] Bar Y. Diamant I. Wolf L. Lieberman S. Konen E. Greenspan H. (2015). “Chest pathology detection using deep learning with non-medical training,” in 2015 IEEE 12th International Symposium on Biomedical Imaging (ISBI) (Brooklyn, NY: IEEE), 294–297. 10.1109/ISBI.2015.7163871

[B4] Bhatnagar G. Wu Q. J. Liu Z. (2013). Directive contrast based multimodal medical image fusion in nsct domain. IEEE Trans. Multimedia 15, 1014–1024. 10.1109/TMM.2013.2244870

[B5] Bhatnagar G. Wu Q. J. Liu Z. (2015). A new contrast based multimodal medical image fusion framework. Neurocomputing 157, 143–152. 10.1016/j.neucom.2015.01.025

[B6] Chen Q. Xu J. Koltun V. (2017). “Fast image processing with fully-convolutional networks,” in Proceedings of the IEEE International Conference on Computer Vision (Venice), 2497–2506. 10.1109/ICCV.2017.273

[B7] Chen Y. Yang M. Chen X. Liu B. Wang H. Wang S. (2018). Sensorineural hearing loss detection via discrete wavelet transform and principal component analysis combined with generalized eigenvalue proximal support vector machine and tikhonov regularization. Multimedia Tools Appl. 77, 3775–3793. 10.1007/s11042-016-4087-6

[B8] Du J. Li W. Lu K. Xiao B. (2016). An overview of multi-modal medical image fusion. Neurocomputing 215, 3–20. 10.1016/j.neucom.2015.07.160

[B9] Everingham M. Van Gool L. Williams C. K. I. Winn J. Zisserman A. (2012). The PASCAL Visual Object Classes Challenge 2012 (VOC2012) Results. Available online at: http://www.pascal-network.org/challenges/VOC/voc2012/workshop/index.html

[B10] Fei Y. Wei G. Zongxi S. (2017). Medical image fusion based on feature extraction and sparse representation. Int. J. Biomed. Imaging 2017, 1–13. 10.1155/2017/3020461 28321246 PMC5339635

[B11] Geng P. Sun X. Liu J. (2017). Adopting quaternion wavelet transform to fuse multi-modal medical images. J. Med. Biol. Eng. 37, 230–239. 10.1007/s40846-016-0200-6 29755307 PMC5928192

[B12] Guo X. Nie R. Cao J. Zhou D. Qian W. (2018). Fully convolutional network-based multifocus image fusion. Neural Comput. 30, 1775–1800. 10.1162/neco_a_01098 29894654

[B13] Han Y. Cai Y. Cao Y. Xu X. (2013). A new image fusion performance metric based on visual information fidelity. Inform. Fusion 14, 127–135. 10.1016/j.inffus.2011.08.002

[B14] Hariharan B. Arbeláez P. Bourdev L. Maji S. Malik J. (2011). “Semantic contours from inverse detectors,” in 2011 International Conference on Computer Vision (Barcelona: IEEE), 991–998. 10.1109/ICCV.2011.6126343

[B15] Huang B. Yang F. Yin M. Mo X. Zhong C. (2020). A review of multimodal medical image fusion techniques. Comput. Math. Methods Med. 2020, 1–16. 10.1155/2020/8279342 32377226 PMC7195632

[B16] James A. P. Dasarathy B. V. (2014). Medical image fusion: a survey of the state of the art. Inform. Fusion 19:4–19. 10.1016/j.inffus.2013.12.002

[B17] Kessy A. Lewin A. Strimmer K. (2018). Optimal whitening and decorrelation. Am. Stat. 72, 309–314. 10.1080/00031305.2016.1277159

[B18] Lewis J. J. O'Callaghan R. J. Nikolov S. G. Bull D. R. Canagarajah N. (2007). Pixel-and region-based image fusion with complex wavelets. Inform. Fusion 8, 119–130. 10.1016/j.inffus.2005.09.006

[B19] Li H. He X. Tao D. Tang Y. Wang R. (2018). Joint medical image fusion, denoising and enhancement via discriminative low-rank sparse dictionaries learning. Pattern Recogn. 79, 130–146. 10.1016/j.patcog.2018.02.005

[B20] Li H. Manjunath B. Mitra S. K. (1995). Multisensor image fusion using the wavelet transform. Graph. Models Image Process. 57, 235–245. 10.1006/gmip.1995.1022

[B21] Li H. Wu X.-J. (2018). Infrared and visible image fusion using latent low-rank representation. *arXiv [Preprint]. arXiv:1804.08992*. Available online at: https://arxiv.org/abs/1804.08992

[B22] Li S. Kang X. Hu J. (2013). Image fusion with guided filtering. IEEE Trans. Image Process. 22, 2864–2875. 10.1109/TIP.2013.2244222 23372084

[B23] Li Y. Sun Y. Huang X. Qi G. Zheng M. Zhu Z. (2018). An image fusion method based on sparse representation and sum modified-laplacian in nsct domain. Entropy 20:522. 10.3390/e20070522 33265611 PMC7513046

[B24] Liu G. Yan S. (2011). “Latent low-rank representation for subspace segmentation and feature extraction,” in 2011 International Conference on Computer Vision (Barcelona: IEEE), 1615–1622. 10.1109/ICCV.2011.6126422

[B25] Liu Y. Chen X. Cheng J. Peng H. (2017). “A medical image fusion method based on convolutional neural networks,” in 2017 20th International Conference on Information Fusion (Fusion) (Xi'an: IEEE), 1–7. 10.23919/ICIF.2017.8009769

[B26] Liu Y. Chen X. Ward R. K. Wang Z. J. (2016). Image fusion with convolutional sparse representation. IEEE Signal Process. Lett. 23, 1882–1886. 10.1109/LSP.2016.2618776

[B27] Liu Y. Chen X. Ward R. K. Wang Z. J. (2019). Medical image fusion via convolutional sparsity based morphological component analysis. IEEE Signal Process. Lett. 26, 485–489. 10.1109/LSP.2019.2895749

[B28] Liu Y. Wang Z. (2014). Simultaneous image fusion and denoising with adaptive sparse representation. IET Image Process. 9, 347–357. 10.1049/iet-ipr.2014.0311

[B29] Long J. Shelhamer E. Darrell T. (2015). “Fully convolutional networks for semantic segmentation,” in Proceedings of the IEEE Conference on Computer Vision and Pattern Recognition (Boston, MA), 3431–3440. 10.1109/CVPR.2015.7298965 27244717

[B30] Lu S. Lu Z. Zhang Y.-D. (2019). Pathological brain detection based on alexnet and transfer learning. J. Comput. Sci. 30, 41–47. 10.1016/j.jocs.2018.11.008

[B31] Lu S. Wang S.-H. Zhang Y.-D. (2020). Detection of abnormal brain in MRI via improved Alexnet and ELM optimized by chaotic bat algorithm. Neural Comput. Appl. 32, 1–13. 10.1007/s00521-020-05082-4

[B32] Manchanda M. Sharma R. (2018). An improved multimodal medical image fusion algorithm based on fuzzy transform. J. Visual Commun. Image Represent. 51, 76–94. 10.1016/j.jvcir.2017.12.011

[B33] Mertens T. Kautz J. Van Reeth F. (2009). “Exposure fusion: a simple and practical alternative to high dynamic range photography,” in Computer Graphics Forum, Vol. 28, eds R. Scopigno and E. Gröller (Wiley Online Library), 161–171. 10.1111/j.1467-8659.2008.01171.x

[B34] Petrovic V. S. Xydeas C. S. (2004). Gradient-based multiresolution image fusion. IEEE Trans. Image Process. 13, 228–237. 10.1109/TIP.2004.823821 15376943

[B35] Razzak M. I. Naz S. Zaib A. (2018). “Deep learning for medical image processing: overview, challenges and the future,” in Classification in BioApps, eds N. Dey, A. Ashour, and S. Borra (Cham: Springer), 323–350. 10.1007/978-3-319-65981-7_12

[B36] Toet A. (1989). A morphological pyramidal image decomposition. Pattern Recogn. Lett. 9, 255–261. 10.1016/0167-8655(89)90004-4

[B37] Vidoni E. D. (2012). The whole brain atlas: www.med.harvard.edu/aanlib/. J. Neurol. Phys. Therapy 36:108 10.1097/NPT.0b013e3182563795

[B38] Walrand S. Hesse M. Jamar F. (2017). “SPECT/CT, PET/CT and PET/MR principles,” in Diagnostic and Therapeutic Nuclear Medicine for Neuroendocrine Tumors, eds K. Pacak and D. Taïeb (Cham: Springer), 163–200. 10.1007/978-3-319-46038-3_8

[B39] Wang L. Ouyang W. Wang X. Lu H. (2015). “Visual tracking with fully convolutional networks,” in Proceedings of the IEEE International Conference on Computer Vision (Santiago), 3119–3127. 10.1109/ICCV.2015.357

[B40] Wang P. Zhang C. Cai S. Li L. (2013). Accelerated matrix recovery via random projection based on inexact augmented lagrange multiplier method. Trans. Tianjin Univ. 19, 293–299. 10.1007/s12209-013-2135-0

[B41] Wang Q. Li S. Qin H. Hao A. (2015). Robust multi-modal medical image fusion via anisotropic heat diffusion guided low-rank structural analysis. Inform. Fusion 26, 103–121. 10.1016/j.inffus.2015.01.001

[B42] Wang S. Yang M. Du S. Yang J. Liu B. Gorriz J. M. (2016). Wavelet entropy and directed acyclic graph support vector machine for detection of patients with unilateral hearing loss in mri scanning. Front. Comput. Neurosci. 10:106 10.3389/fncom.2016.00106 27807415 PMC5069288

[B43] Wang S. Yang M. Li J. Wu X. Wang H. Liu B. (2017). Texture analysis method based on fractional fourier entropy and fitness-scaling adaptive genetic algorithm for detecting left-sided and right-sided sensorineural hearing loss. Fundament. Inform. 151, 505–521. 10.3233/FI-2017-1507

[B44] Xydeas C. Petrovic V. (2000). Objective image fusion performance measure. Electron. Lett. 36, 308–309. 10.1049/el:20000267

[B45] Yin M. Liu X. Liu Y. Chen X. (2018). Medical image fusion with parameter-adaptive pulse coupled neural network in nonsubsampled shearlet transform domain. IEEE Trans. Instrument. Meas. 68, 49–64. 10.1109/TIM.2018.2838778

[B46] Zhang Q. Guo B. L. (2009). Multifocus image fusion using the nonsubsampled contourlet transform. Signal Process. 89, 1334–1346. 10.1016/j.sigpro.2009.01.012

[B47] Zhang Y. Ranjan Nayak D. Yang M. Yuan T.-F. Liu B. Lu H.. (2017). Detection of unilateral hearing loss by stationary wavelet entropy. CNS Neurol. Disord. Drug Targets 16, 122–128. 10.2174/1871527315666161026115046 27784224

[B48] Zhao W. Lu H. (2017). Medical image fusion and denoising with alternating sequential filter and adaptive fractional order total variation. IEEE Trans. Instrument. Meas. 66, 2283–2294. 10.1109/TIM.2017.2700198

[B49] Zhu Z. Zheng M. Qi G. Wang D. Xiang Y. (2019). A phase congruency and local laplacian energy based multi-modality medical image fusion method in nsct domain. IEEE Access 7, 20811–20824. 10.1109/ACCESS.2019.2898111

